# Analysis of alcohol dependence phenotype in the COGA families using covariates to detect linkage

**DOI:** 10.1186/1471-2156-6-S1-S143

**Published:** 2005-12-30

**Authors:** Brian H Reck, Nandita Mukhopadhyay, Hui-Ju Tsai, Daniel E Weeks

**Affiliations:** 1 Department of Human Genetics, Graduate School of Public Health, University of Pittsburgh, Pittsburgh, PA 15261 USA; 2 University of California, San Francisco, San Francisco, CA 94143 USA; 3 Department of Biostatistics, Graduate School of Public Health, University of Pittsburgh, Pittsburgh, PA 15261 USA; 4 Lung Biology Center, San Francisco General Hospital, San Francisco, CA 94110 USA

## Abstract

Linkage analysis methods that incorporate etiological heterogeneity of complex diseases are likely to demonstrate greater power than traditional linkage analysis methods. Several such methods use covariates to discriminate between linked and unlinked pedigrees with respect to a certain disease locus. Here we apply several such methods including two mixture models, ordered subset analysis, and a conditional logistic model to genome scan data on the DSM-IV alcohol dependence phenotype on the Collaborative Studies on Genetics of Alcoholism families, and compare the results to traditional nonparametric linkage analysis. In general, there was little agreement among the various covariate-based linkage statistics. Linkage signals with empirical *p*-values less than 0.001 were detected on chromosomes 3, 4, 7, 10, and 12, with the highest peak occurring at the GABRB1 gene using the ecb21 covariate.

## Background

Etiological heterogeneity is inevitable when large sets of pedigree data are analyzed for complex diseases, where the susceptibility loci may vary from one pedigree to another. Such heterogeneity, if unrecognized, tends to reduce the power to detect linkage. Covariate-based methods attempt to adjust for heterogeneity by using covariate data to discriminate between pedigrees with different disease etiologies; however, since these methods are relatively new, few studies have applied them to real datasets [1-3]. The most comprehensive investigation comparing these methods is an extensive simulation under different gene × environment interaction models performed by Tsai [4]. The Collaborative Studies on Genetics of Alcoholism (COGA) [5] family dataset provides an opportunity to apply covariate-based methods because it contains several biologically meaningful covariates of the alcoholism phenotype.

In this study, we applied four covariate-based methods to the COGA families from the Genetic Analysis Workship 14 dataset. Our aim was to identify new genes responsible for alcoholism, as well as to study whether previously detected regions of linkage were also detected using these new methods. The methods included the pre-cluster and covariate-identity by descent (cov-IBD) models of Devlin et al. [6], ordered subset analysis (OSA) of Hauser et al. [7], and the conditional logistic regression model of Olson [8] implemented within the LODPAL program of S.A.G.E. [9]. The results were compared to traditional nonparametric linkage analysis using GENEHUNTER-PLUS [10]. We used simulation to estimate the significance of our linkage signals empirically.

## Methods

### Covariate-based linkage analysis methods

One class of models assumes that a proportion of the pedigrees are linked to the disease gene, while the remaining pedigrees are affected due to some other reason. Membership in the linked group is predicted using one or more covariates assumed to be related to the disease. The pre-cluster, cov-IBD, and OSA models fall into this category. Regression-based models that condition on the covariate values are a second category of heterogeneity-based methods, Olson's method being an example.

Pre-cluster and cov-IBD by Devlin et al. are mixture models that analyze affected sib-pair (ASP) data [6]. Each ASP is assigned a pair-specific covariate value. Linkage at a marker is detected by maximizing the likelihood as a function of the probability, α, of each sib pair being in the linked group and its IBD proportions. Pre-cluster determines α by clustering on the covariates prior to testing for excess IBD sharing, while cov-IBD uses both the covariates and IBD information to determine α while simultaneously testing for linkage.

OSA determines the ordered subset of the pedigrees that provides maximal evidence for linkage [11]. Each pedigree is assigned an overall pedigree-level covariate value, and pedigrees are then ranked in increasing or decreasing order of their covariate values. The OSA statistic is the maximum of the LOD scores over the ordered subsets. The advantage of OSA is that *a priori *specification of the linked and unlinked subsets is not required; however, it ignores the magnitude of the covariate values, considering only the rank.

Olson's method uses a conditional-logistic representation of an affected relative pair (ARP) likelihood ratio that includes the effects of covariates as additional parameters in a test for linkage [8]. This model allows for the inclusion of pair-wise covariates and is valid for any type of ARP. The model assumes a multiplicative effect of the covariate on the genetic relative risk, and can be used to test whether the covariate contributes significant information about linkage in a region where linkage is known to exist.

### Phenotypes and covariates

The DSM-IV alcohol dependence phenotype (ALDX2) [12] was recoded into a binary disease phenotype. Subjects having the affected phenotype were maintained as affected, those having no information were recoded as unknown, and everyone else with a known phenotype was coded as unaffected.

We selected four quantitative phenotypes including two electrophysiological measurements as possible covariates: 1) age of onset for alcohol dependence, 2) number of packs of cigarettes per day for a year, CIGPKYRS, 3) Visual Oddball experiment data for the target case from the far frontal left side channel, ttth1 and 4) data from the Eyes Closed Resting electroencephalogram experiment, ecb21. Age of onset and ecb21 were selected because they divided up the affected sib pairs into noticeable clusters (data not shown), which is necessary for the cov-IBD and pre-clustering methods to work well [4]. Clustering was performed using the *mclust *[13,14] function of R. The ttth1 phenotype was selected because it has been linked to known regions on the genome [15,16] on the COGA families. The CIGPKYRS phenotype was selected as evidence of tendency to substance abuse.

For pre-cluster, *mclust *was used to cluster the set of affected sibling-pairs simultaneously on two dimensions: minimum of affected's phenotype and maximum of affected's phenotype, over the entire pedigree containing that pair. Before clustering, we standardized each set of covariate values by subtracting the mean and dividing by the standard deviation of the sample in order to enhance numerical stability. By our clustering scheme, membership of each pedigree to either cluster is determined by X_ped_, where:

X_ped _= √[(min of affected's phenotypes)^2 ^+ (max of affected's phenotypes)^2^].

The two clusters were designated as **G1 **and **G2**, based on the Euclidian distance of their centres from the origin, **G1 **representing the nearer cluster. Because OSA allows for only one covariate per pedigree, we assigned X_ped _values as pedigree-level covariates prior to running OSA. We ran LODPAL on affected sib pairs using both the sum and the difference of each pair's covariate values, reporting the best score. The multiple-testing issue arising in this case was taken care of by the empirical *p*-value calculation.

### Linkage analysis

We used all 143 multiplex pedigrees and the 315 microsatellite markers located on chromosomes 1 through 22. Our analysis was not appropriate for X-linked data. Due to software limitations, the seven largest pedigrees were broken into smaller components or trimmed of uninformative individuals, resulting in 156 pedigrees overall. Multipoint IBD probabilities were obtained using MERLIN version 0.10.2 [5] for use within LODPAL and pre-cluster at each marker, and four equally spaced intermediate positions. Multipoint nonparametric linkage analysis was peformed using GENEHUNTER-PLUS based on the S_all _statistic. MEGA2 [17] was used to set up files for MERLIN, GENEHUNTER-PLUS, and LODPAL.

For pre-cluster, we computed two likelihoods: with **G1 **as the linked cluster, and with **G2 **as the linked cluster. Similarly, for OSA, we used both orderings of the X_ped _values: **L2H**, ordered small to large so that linked pedigrees have smaller covariate values; **H2L**, ordered large to small, so linked pedigrees have larger covariate values. Marker positions are reported by using Haldane map function. The chromosomal locations of the genes that were not included in the COGA marker map were obtained from the Marshfield web site and converted to Haldane map distances.

### Empirical significance

A small region on chromosome 7 spanning 27–61 cM was selected for determining the empirical significance of LOD scores obtained from the various covariate methods. We simulated 1,000 replicates of the genotype data using SIMULATE [18] under the hypothesis of no linkage while keeping the pedigrees and covariates constant. The genotype data were then analyzed by each method, with each of the four covariates. The simulated LOD scores for all of the markers were pooled to create the empirical null distributions for each covariate and method. The validity of pooling markers is discussed in [4].

## Results

The NPL analysis produced a single peak with LOD score 2.68 at D10S544 on chromosome 10. We have not reported cov-IBD results because these were not significantly different from the pre-cluster results. Table 1 contains the top three significant results for pre-cluster, OSA, and LODPAL. OSA produced elevated LOD scores for all covariates in the region found by nonparametric linkage analysis as did pre-cluster using the ttth1 covariate (results not shown). The highest peak for LODPAL is at the GABRB1 gene that has been identified previously as being linked to alcoholism [15]. The OSA peak at D7S2846 is within 22 cM of the NPY2 gene, and the peak on chromosome 11 for the pre-cluster model lies within 20 cM of the DRD2 gene. Although association between specific variants of the DRD2 gene and alcoholism has been noted previously, no linkage study has detected alcoholism genes in this region. LODPAL found a suggestive linkage peak on chromosome 6 at 142 cM with LOD score 3.09 using the age on onset as covariate, which is close to the ALDH8A1 gene, as well as the GRK1 gene.

**Figure 1:**
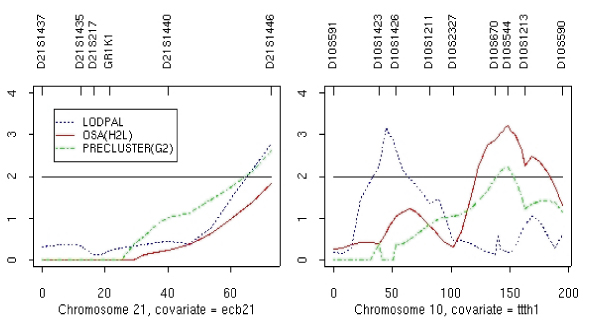
Plot of -log10(*p*-value) showing examples of agreement and disagreement between the three covariate based methods.

Except for one region on chromosome 21 (Figure 1), which showed consistently elevated LOD scores for all methods using the ecb21 covariate, there were no peaks in common across methods. Using the ttth1 covariate, chromosome 10 showed elevated LOD scores for all three methods, but in different regions (Figure 1). There was little commonality between subsets produced by OSA and the linked clusters produced by pre-cluster, for the six peaks listed in Table 1 for these two methods, or for the chromosome 10 peak (comparisons not shown). The 99^th ^percentiles of the empirical null distribution of LOD scores for pre-cluster range between 1.17 and 1.34 for the four covariates; 99^th ^percentiles for OSA are between 1.86 and 2.09; LODPAL's 99^th ^percentile range from 1.99 to 2.24.

## Discussion

Our covariate selection was rather heuristic, based on evidence from clustering, rather than biological reasons. Ideal candidates for covariate statistics would be risk factors with a gene × environment interaction effect and identifying such factors requires prior biological knowledge. A purely environmental risk factor would act as a confounder, reducing the power of the mixture model because it cannot cluster families into linked and unlinked groups. However, it is a challenging issue to determine which of the above classes a covariate falls into, and this bears further investigation within a systematic framework. We would also expect that the choice of the function for creating pedigree-level covariates from individual values would have an effect on the analysis. Indeed, when we used the mean value of the affecteds instead of our X_ped _values, LOD scores were noticeably lower (results not shown). The lack of agreement among the results may be also be due to the sensitivity of the covariate-based methods to the relationship between the covariate and trait under study.

Tsai [4] observed previously that the thresholds for significance tend to be greater for the conditional-logistic model than for the mixture model (1.7 vs. 1.2 for at the 0.01 level). Our investigation supports her observations, although the conditional-logistic model threshold appeared to be higher than her findings. Because the theoretical distributions for the test statistics of the conditional logistic model, OSA, and cov-IBD are approximations, in order to make direct comparisons between the methods we recommend using an empirical distribution of the LOD scores.

## Abbreviations

ARP: Affected relative pair

ASP: Affected sib pair

COGA: Collaborative Study on the Genetics of Alcoholism

cov-IBD: Covariate identity by descent

IBD: Identity by descent

OSA: Ordered subset analysis

## Authors' contributions

BHR, NM, and H-JT contributed equally to the data processing, analysis, and the writing of the manuscript. DEW contributed to the design of the study and writing of the manuscript.

## Figures and Tables

**Table 1:** Most significant peaks for LODPAL, OSA, and pre-cluster

Chr	Position (cM)	Locus	Covariate	Linked cluster/subset and size^a^	LOD score	-log_10 _(*p*-value)
LODPAL						
3	160.00	ATA34G06		NA	4.72	>4.48
4	61.00	GABRB1	ecb21	NA	6.61	>4.48
12	187.00	D12S1045	ttth1	NA	4.26	>4.48
OSA						
4	p-ter	D4S2366	ttth1	H2L [42 out of 156 ]	2.48	2.44
7	62.19	D7S2846	Age of onset	H2L [35 out of 156]	3.09	3.30
10	148.19	D10S544	ttth1	H2L [41 out of 156]	3.40	3.32
Pre-cluster						
10	194.37	D10S590	ecb21	G2 [101 out of 142]	2.10	2.89
11	90.91	D11S2002	ttth1	G1 [36 out of 133]	1.90	2.68
21	73.68	D21S1446	ecb21	G2 [108 out of 149]	1.86	2.62
GENEHUNTER-PLUS						
10	148.19	D10S544	NA	NA	2.86	>4.48

^a^The numbers within square brackets in the "Linked cluster/subset" column represent the cluster or subset size in terms of ASPs for pre-cluster and pedigrees for OSA.
